# Repeated Low Intensity Blast Exposure Is Associated with Damaged Endothelial Glycocalyx and Downstream Behavioral Deficits

**DOI:** 10.3389/fnbeh.2017.00104

**Published:** 2017-06-09

**Authors:** Aaron A. Hall, Mirian I. Mendoza, Hanbing Zhou, Michael Shaughness, Richard M. McCarron, Stephen T. Ahlers

**Affiliations:** Neurotrauma Department, Operational and Undersea Medicine, Naval Medical Research Center, Silver Spring MD, United States

**Keywords:** blast, glycocalyx, behavioral deficits, traumatic brain injury, repeated low intensity

## Abstract

Current clinical research into mild traumatic brain injury (mTBI) has focused on white matter changes as identified by advanced MRI based imaging techniques. However, perivascular tau accumulation in the brains of individuals diagnosed with mTBI suggests that the vasculature plays a key role in the pathology. This study used a rat model to examine whether the endothelial glycocalyx, a layer of the vasculature responsible for sensing luminal shear forces, is damaged by exposure to repeated low intensity blast, and whether this layer is associated with observed behavioral deficits. The blast exposure used consisted of 12, 40 kPa blast exposures conducted with a minimum of 24 h between blasts. We found that repeated blast exposure reduced glycocalyx length and density in various brain regions indicating damage. This blast exposure paradigm was associated with a mild performance decrement in the Morris water maze (MWM) which assesses learning and memory. Administration of hyaluronidase, an enzyme that binds to and degrades hyaluronan (a major structural component of the glycocalyx) prior to blast exposure reduced the observed behavioral deficits and induced a thickening of the glycocalyx layer. Taken together these findings demonstrate that the endothelial glycocalyx degradation following repeated blast is associated with behavioral decrements which can be prevented by treatment with hyaluronidase.

## Introduction

Military personnel experience repeated low intensity blast exposure during training and/or combat, often resulting in mTBI. By definition mTBI patients lack overt signs of trauma such as hemorrhage or swelling. Advanced imaging techniques have identified changes in white matter tracts; however, the underlying pathology is still poorly understood. Post-mortem brain samples from mTBI patients showed significant perivascular (or intraneuronal) accumulation of phosphorylated Tau, indicating a vascular component to this disease state (Goldstein et al., 2012). Evidence from rodent models of blast exposure demonstrates functional alterations in cerebrovasculature to include blood-brain barrier (BBB) disruption and increased intracranial pressure (ICP) (Saljo et al., 2010, 2011). The major contributor to these vascular changes is currently thought to be either increased hydrostatic pressures induced by the blast wave impinging on the major blood vessels in the thorax and neck, negative pressure waves associated with skull flexure (Bolander et al., 2011), or direct interaction of the blast wave with brain tissue (Cernak, 2010; Cernak and Noble-Haeusslein, 2010; Chavko et al., 2011). Nevertheless, these studies have not identified a discrete component of the vasculature which, when damaged by the primary blast wave or secondary hydrostatic pulse wave, results in downstream cognitive and/or behavioral deficits.

The endothelial glycocalyx is a matrix of heavily glycosylated proteins on the lumen of the vasculature critical to endothelial health. In addition to regulating solute diffusion, immune cell attachment, and complement activation, a primary physiological role of the glycocalyx is to detect changes in luminal shear stress. This shear stress detection role of the glycocalyx is integral to cerebrovascular regulation and damage to the endothelial glycocalyx causes persistent changes in baseline cerebral blood flow (Vogel et al., 2000). As blast exposure likely induces supra-physiological shear stress, we examined the effects of blast exposure on the endothelial glycocalyx. To accomplish this, hyaluronidase, an enzyme that binds to and degrades hyaluronan, was used to explore the link between glycocalyx integrity and downstream behavioral function. We hypothesized that removal of damaged glycocalyx by administration of during the blast paradigm would stimulate regeneration or repair of the glycocalyx and alter downstream behavioral performance.

## Materials and Methods

### Animals

Adult male Long Evans hooded rats (250–350 g; 10–12 weeks of age; Charles River Laboratories International, Inc., Wilmington, MA, United States) were used. All studies were approved by the Institutional Animal Care and Use Committee of the Naval Medical Research Center, and were conducted in conformance with Public Health Service policy on the humane care and use of laboratory animals and the National Institutes of Health (NIH) Guide for the Care and Use of Laboratory Animals.

### Experimental Design

There were two experiments conducted for this study.

*Experiment 1* was conducted to determine the effects of blast on glycocalyx structure. For this experiment rats (*n* = 30, 15/group) were randomly assigned to receive either blast or sham blast exposure as described in the Blast Overpressure Exposure section. Twenty-four hours after the last blast, the rats were then behaviorally assessed using the Morris Water Maze (MWM). The rats were then perfuse/fixed for electron microscopy 48 h following the last blast. During the course of the study two rats did not complete the blast paradigm (due to placement in the wrong group during the blast sequence) and were excluded from the study yielding 28 rats which completed the study with *n* = 14 rats per group for the analysis.

*Experiment 2* was conducted on a separate set of rats to determine the effects of glycocalyx integrity on MWM performance by using hyaluronidase to modify the glycocalyx in rats exposed to blast or sham blast. For this experiment rats (*n* = 40, 10/group) were assigned to four groups: Sham Blast/Saline infusion (SC), Sham Blast/Hyaluronidase infusion (HC), Blast/Saline infusion (SB), Blast/Hyaluronidase infusion (HB). Twenty-four hours after the last blast (or Sham Blast), the rats were then behaviorally assessed using the MWM. The rats were then perfuse/fixed for electron microscopy 48 h following the last blast. During the course of the study six rats were euthanized and excluded due to complications associated with repeated tail vein injections (necrosis/infection), yielding 34 rats which completed the study with 8 or 9 rats per group for the analysis as follows: SC (*n* = 8), HC (*n* = 8), SB (*n* = 9), and HB (*n* = 9).

### Blast Overpressure Exposure

Rats were exposed to blast overpressure using the Walter Reed Army Institute of Research (WRAIR) shock tube as described previously. The shock tube has a 305 mm circular diameter and is a steel tube comprised of a 0.76 m compression chamber that is separated from a 5.18 m expansion chamber. The compression and expansion chambers were separated by (0.05 or 0.075 mm) polyethylene Mylar^TM^ sheets (DuPont Co., Wilmington, DE, United States) that result in a peak pressure wave of (36.45 ± 2.32 kPa), with a mean duration of (3.78 ± 0.09 ms) and an impulse of (80.92 ± 4.41 kPa^∗^ms); mean ± standard error for *n* = 12 similar to previous reports (Ahlers et al., 2012).

**FIGURE 1:**
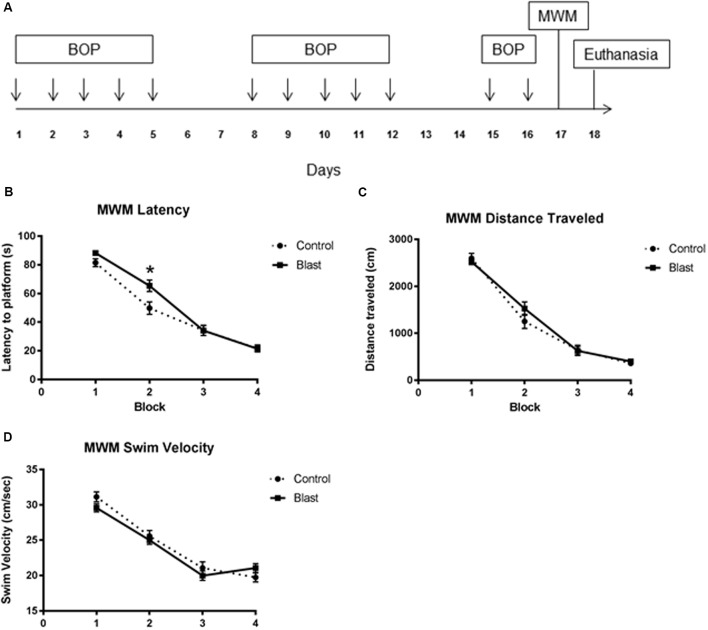
Repeated blast exposure caused performance decrements in the Morris Water Maze. Schema representing the experimental design for experiment 1 **(A)**. Each arrow represents an individual blast overpressure exposure (BOP). Line graphs represent mean MWM trial performance over four consecutive blocks of trials. Each block consisted of four swim trials. Blast exposed animals (solid line) had significantly increased latency to platform **(B)** when compared to controls (dashed line) (52.3 s vs. 46.8 s, two-way ANOVA, *p* = 0.0418). Swim velocity (24.38 cm/s vs. 23.91 cm/s, two-way ANOVA, *p* = 0.3359) **(D)** and Distance traveled (1214 cm vs. 1269 cm, two-way ANOVA, *p* = 0.4443) **(C)** were not significantly different between groups.

Individual rats were anesthetized using 5% isoflurane mixture in air for 2 min. Rats were then put into a cone-shaped plastic restraint device and then placed in the shock tube. Movement was further restricted during the blast exposure using 1.5 cm diameter flattened rubber tourniquet tubing. Three tourniquets were spaced evenly to secure the head region, the upper torso, and the lower torso, while the animal was in the restraint cone. The end of each tube was threaded through a toggle and run outside of the exposure cage, where it was tied to firmly affix the animal and prevent movement during the blast overpressure exposure without restricting breathing. One exposure per day was administered for 12 days over 2.5 weeks according to the schema described in **Figure 1A**. Sham-exposed animals were treated identically except that they were not exposed to blasts.

### Morris Water Maze Testing

The MWM apparatus consisted of a blue, circular tank (74.5 cm deep; 180 cm diameter) filled with water (20°C) to a depth of 60 cm located in a dimly lit room. A platform was submerged to a depth of 1 cm and placed approximately 35 cm from the wall of the pool in the center of the northeast quadrant. The position of the platform remained constant throughout all experiments. Blast or sham exposed rats were given four 4-block trials on a single test day 24 h following the final blast exposure. At the start of each trial, the rat was placed in the pool (snout facing the pool-wall) at one of four equally spaced starting positions: north (N), south (S), east (E), and west (W). The order of the start position was randomized. Each rat was allowed to swim freely in the pool until it found the hidden platform or until 90 s elapsed. If the rat did not find the platform in 90 s it was manually guided to it. Once the rat found the platform it was allowed to rest for 10 s (reinforcement), removed from the pool, gently towel-dried, and placed in a holding cage (warmed by a circulating water pad) in between trials. The dependent measures for this task were (1) latency to reach the platform and (2) length of path (distance swum) to the platform, averaged over the blocked trials. Spatial learning was assessed using the Noldus EthoVision XT (Noldus, Inc, Leesburg, VA, United States) video-tracking system from a camera suspended above the water maze.

### Tail Vein Infusion

Rat tail vein access was achieved using 24 gauge intravenous catheters (SURFLASH, Terumo Medical Corporation, Somerset, NJ, United States). Animals were placed in a plastic restraint and the tails were washed in warm water to promote vasodilation of the tail vein. The injection site was then cleaned with 70% isopropyl alcohol prior to catheter placement. Infusions of saline or Hyaluronidase (2000 IU/kg), (Sigma–Aldrich) were administered as 0.5 ml bolus infusions followed by a 1.0 ml flush with sterile saline. The infusions were given prior to blast every 72 h over the course of the blast exposure sequence (days 1, 4, 7, and 10). To our knowledge, using hyaluronidase to modify brain endothelial glycocalyx has not been previously studied. The dose of hyaluronidase chosen was based on previous studies of enzymatic modification of the glycocalyx in muscle (500 IU) and kidney (1105 IU) in rats (Eskens et al., 2013; Sverrisson et al., 2014). The 2000 IU/kg dose results in a total dose (500–700 IU) which is within the range of doses shown to have effects in different tissue beds and was adjusted for the weight of the animal.

### Perfusion/Fixation and Tissue Harvesting

Rats were injected with a lethal dose (0.5 ml) of Barbituric acid (Euthasol, Virbac Corporation, St. Louis, MO, United States) 24 h following MWM assessment. The animals were then perfused and fixed using the methods described by van den Berg et al. (2003). Briefly, rats were transcardially perfused with 150 ml of cardioplegic solution (in mM: 5.55 Glucose, 114 NaCl, 10 KCl, 1.18 KH_2_PO_4_, 1.17 MgSO_4_, 25 HCO_3_, 5 HEPES, 0.025 EDTA; 0.1%BSA, PH 7.4) followed by 250 ml of fixation buffer (1% glutaraldehyde, 4% Paraformaldehyde, 0.05% Alcian Blue, 30 mM MgCl_2_, in 84 mM H_2_PO_4_, PH7.4) over the course of 40 min. The brain was removed and post-fixed for ≥96 h in fresh fixation buffer, then dissected to remove sections of the hippocampus, cortex, corpus callosum, amygdala, and corpus striatum for electron microscopy processing.

### Electron Microscopy Processing

Tissue samples from each brain region were cut into 1 mm × 1 mm blocks and post fixed in an aqueous solution of 1% osmium tetroxide and 1% lanthanum nitrate for 1 h. The samples were contrast stained en-block with 1% Uranyl acetate for 1 h followed by sequential dehydration in ethanol and propylene oxide. Samples were then embedded in Epon 812 acrylic resin and ultrathin sections of tissue were placed on copper grids for imaging with a JEOL 100CXII electron microscope. Capillaries within the tissue were imaged at a 10,000× magnification. Only capillaries which entirely fit within the image were selected for analysis. Five images per region were captured by a microscopist blinded to the nature of the samples. Film containing the images was developed and digitized using an EPSON V750 pro scanner.

### Image Analysis and Quantification

The digital images were saved as tagged image format files, and then opened in the NIH ImageJ64 program (Rasband, W.S., ImageJ, U. S. National Institutes of Health, Bethesda, MD, United States^1^, 1997–2011) for quantification of the glycocalyx layer and converted to grayscale images. To quantify the glycocalyx, the luminal membrane of the endothelial cell was outlined using the polygon selection tool. All extra-luminal components were then removed using the “clear outside” command. The glycocalyx was then measured by adjusting the threshold function such that the glycocalyx was highlighted. The area of the glycocalyx was then measured by using the “Analyze: Measure” command. The threshold was then reset to highlight the entire lumen of the capillary and measured using the “Analyze: Measure” command. The resulting area value for the glycocalyx was divided by the luminal area and expressed as percent lumen area. For consistency all the images were analyzed by an individual blinded to the nature of the groups.

### Statistical Analysis

Data collected during each experiment was compiled into a spreadsheet (Excel 2010, Microsoft Corporation, Redmond, WA, United States) then exported into downstream statistical analysis software. All statistical analysis was performed using Graphpad Prism (Version 6.0, La Jolla, CA, United States). MWM data from experiment 1 was analyzed by Two-way repeated measures ANOVA with *post hoc* Sidak’s test. MWM data from experiment 2 was analyzed by One-way repeated measures ANOVA with *post hoc* Sidak’s test. Glycocalyx quantification data was analyzed by Two-way ANOVA with *post hoc* Bonferroni’s test.

## Results

### Repeated Blast Exposure Caused Decrements in MWM Performance

Exposing rats to twelve 40 kPa blasts causes acute behavioral decrements in the absence of gross histological signs of injury. This blast exposure paradigm was previously shown to produce significant decrements in latencies to platform which normalized to controls by the final block indicating slower acquisition of the task (Ahlers et al., 2012). To determine whether the endothelial glycocalyx is involved with this behavioral deficit, rats (*n* = 14/group; 28 total) were exposed to an identical blast (or sham blast) exposure paradigm with MWM testing conducted 24 h following the last blast. The testing consisted of four blocks of four trials administered over the course of a single day. As seen previously, (Ahlers et al., 2012) blast-exposed animals had significantly increased latency to platform (**Figure 1B**) when compared to sham blast controls [52.3 s vs. 46.8 s, two-way ANOVA: treatment × time *F*(3,330) = 3.364, *P* = 0.0189; time *F*(3,330) = 185.0, *P* < 0.0001; exposure *F*(1,110) = 4.242, *p* = 0.0418]. Swim velocity [24.38 cm/s vs. 23.91 cm/s, two-way ANOVA: treatment × time *F*(3,278) = 1.700, *P* = 0.1671; time *F*(3,330) = 95.03, *P* < 0.0001; exposure *F*(1,278) = 0.9292, *p* = 0.3359] (**Figure 1D**) and Distance traveled [1214 cm vs. 1269 cm two-way ANOVA: treatment × time *F*(3,278) = 1.113, *P* = 0.3442; time *F*(3,278) = 183.3, *P* < 0.0001; exposure *F*(1,278) = 0.5868, *p* = 0.4443] (**Figure 1C**) were not significantly different between groups.

### The Endothelial Glycocalyx Was Damaged by Repeated Low Intensity Exposure

**FIGURE 2:**
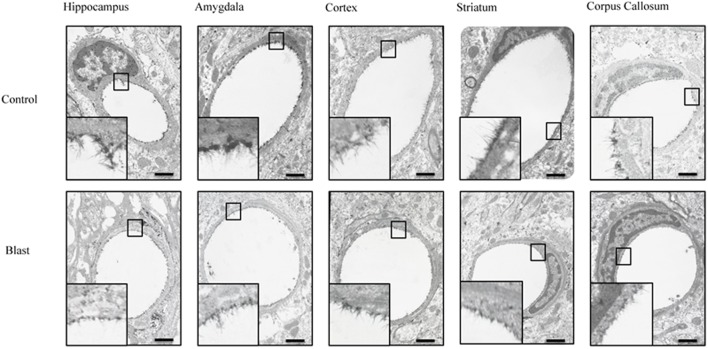
Blast exposure reduced glycocalyx thickness in brain capillaries. Representative electron micrographs of capillaries from hippocampus, amygdala, cerebral cortex, corpus striatum, and corpus callosum taken from blast and sham blasted animals. Endothelial glycocalyx is the darkly stained filamentous layer present on the lumen of the capillary. All insets represent magnified image; scale bar = 500 nm.

**FIGURE 3:**
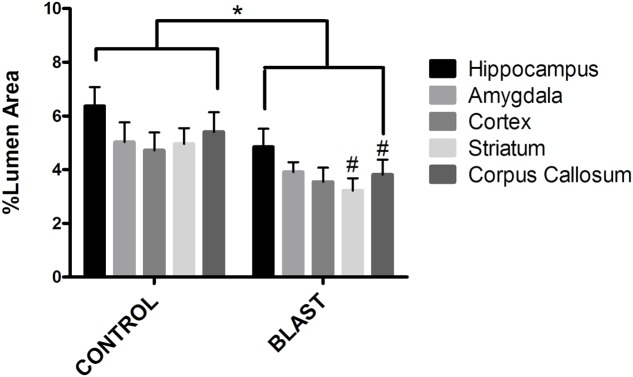
Blast exposure significantly reduced glycocalyx thickness in rats. Mean area of the glycocalyx quantified for each brain region and expressed as a percentage of the total capillary lumen area. Capillaries from blast exposed rats showed a significant reduction in glycocalyx area in the Corpus Callosum (3.811%), Striatum (3.217%), and Hippocampus (4.849%) compared to sham controls (5.403, 4.958, and 6.371%, respectively) Asterisk denotes global significance between sham blast and blast exposed capillaries (5.297% vs. 3.867%, Two way ANOVA, *p* = 0.0003); while pound symbol denotes significance between sham blast and blast exposed capillaries in identical brain regions.

Electron micrographs were prepared of capillaries from the amygdala, hippocampus, cortex, corpus callosum, and striatum brain regions. The endothelial glycocalyx was observed to be a filamentous mesh lining the lumen of the capillary varying in thickness, depending on the brain region similar to that previously observed in other tissue beds. Capillaries imaged from blast exposed animals showed reductions in area of the glycocalyx layer in all five brain regions imaged (**Figure 2**). Quantification of the percent lumen area of the capillary lumen occupied by glycocalyx revealed a global 20% decrease for all brain areas examined in blast exposed animals when compared to controls (**Figure 3**). Interestingly this decrement was uniform, preserving the regional differences in glycocalyx thickness.

### Hyaluronidase Infusion during the Blast Exposures Caused Glycocalyx Thickening in Cortical Microvessels

**FIGURE 4:**
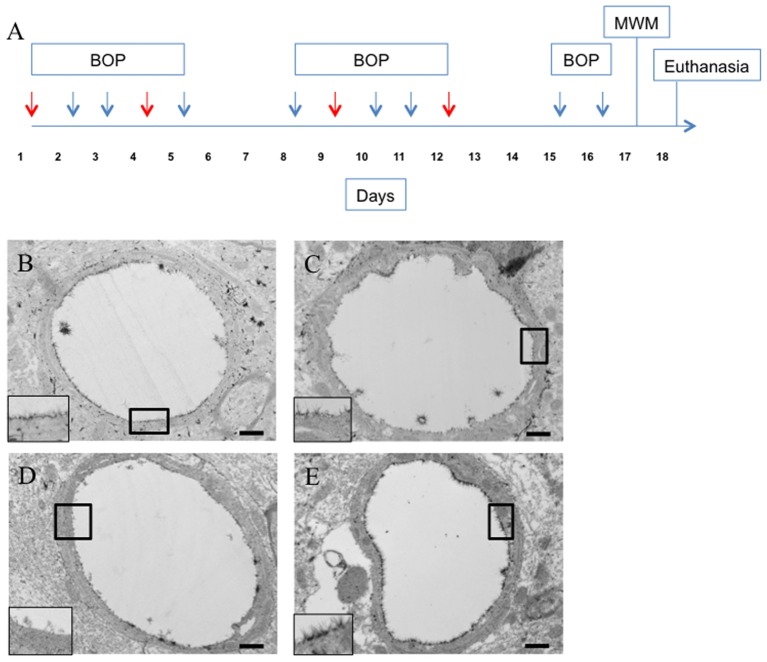
Hyaluronidase infusion increased glycocalyx thickness in sham and blasted rats. Schema representing experimental design for experiment 2 **(A)**. Each arrow represents an individual blast overpressure exposure (BOP) with red arrows denoting days with hyaluronidase infusion. Representative electron micrographs of capillaries from cerebral cortex taken from blast and sham blasted animals infused with hyaluronidase or vehicle. Capillaries from hyaluronidase infused sham controls **(C)** and blast exposed **(E)** rats had qualitatively thicker glycocalyx when compared to saline infused sham controls **(B)** or blast exposed **(D)** animals. All insets represent magnified image; scale bar = 500 nm.

We then examined whether the observed behavioral decrements in the blast exposed animals were related to the observed glycocalyx damage. To accomplish this, rats were blasted according to the same schedule as in experiment 1 and received tail vein infusions of hyaluronidase or saline immediately prior to blast on days 1, 4, 7, and 10 as shown in the schema in **Figure 4A**. Hyaluronidase has a tissue half-life of approximately 19 h in muscle following intravenous injection (Muckenschnabel et al., 1998). The 72 h latency between hyaluronidase infusions was chosen to allow sufficient clearance of systemic hyaluronidase between infusions.

**FIGURE 5:**
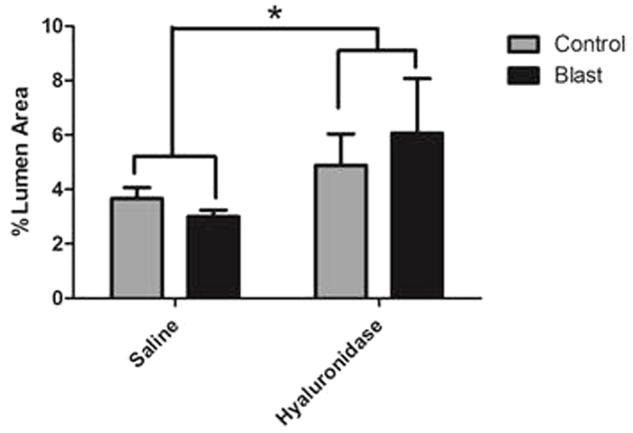
Hyaluronidase infusion significantly increased glycocalyx thickness in sham and blasted rats. Mean area of the glycocalyx quantified from the cerebral cortex of rats from each treatment group and expressed as a percentage of the total capillary lumen area (B). Capillaries from hyaluronidase infused rats showed a significant increase in percent lumen area of the glycocalyx in both sham exposed (HC, 4.886%) and blast exposed (HB, 6.067%) groups when compared to saline infused sham exposed (SC, 3.262%) and blast exposed (SB, 3.517%) groups. Asterisk denotes global significance between saline and hyaluronidase treatment groups (3.389% vs. 5.47%, Two-way ANOVA *p* = 0.0008).

Interestingly, hyaluronidase treated control (C) and blast (E) animals showed marked increases in both length and thickness of glycocalyx when compared to saline treated control (B) or blast (D) animals irrespective of blast exposure (**Figures 4B–E**). Quantification of the glycocalyx area revealed that hyaluronidase treatment significantly increased glycocalyx area in both controls and blast exposed rats when compared to vehicle (saline) treatment. Capillaries from hyaluronidase infused rats showed a significant increase in percent lumen area of the glycocalyx in both sham exposed (HC, 4.886%) and blast exposed (HB, 6.067%) groups when compared to saline infused sham exposed (SC, 3.262%) and blast exposed (SB, 3.517%) groups. There was also a global significant difference between saline and hyaluronidase treatment groups (3.389% vs. 5.47%, Two-way ANOVA *p* = 0.0008) (**Figure 5**).

### Hyaluronidase Infusion Attenuated Performance Decrements in the Morris Water Maze in Blast Exposed Rats

**FIGURE 6:**
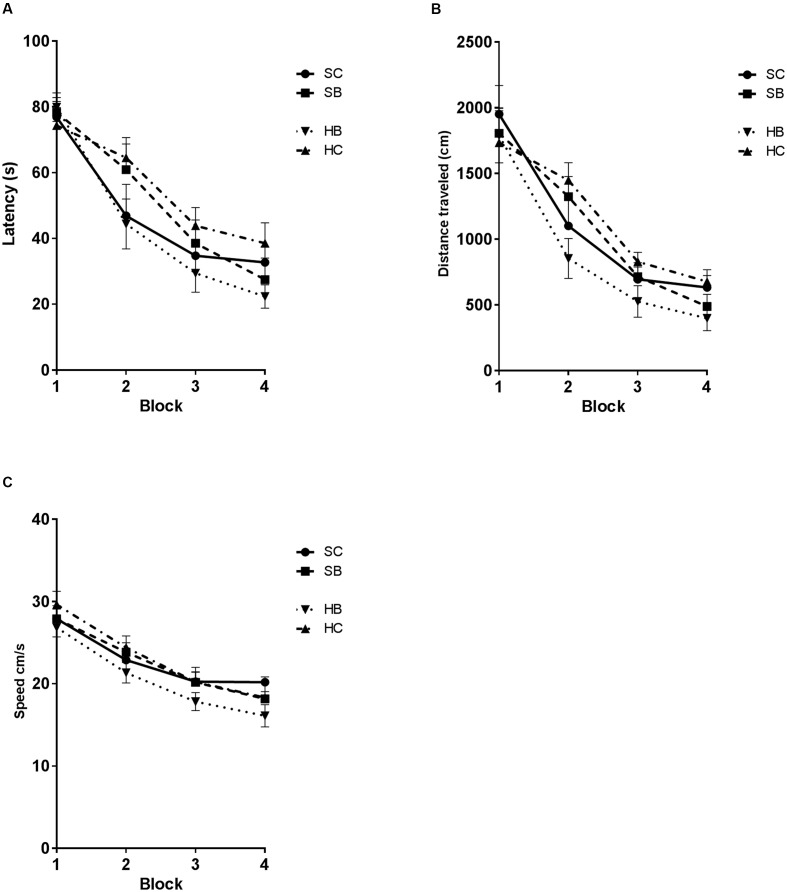
Hyaluronidase infusion attenuated blast induced deficits in the Morris Water maze task. Line graph depicts mean MWM trial latencies over four consecutive blocks of trials **(A)**. Saline infused blast exposed rats (SB) showed an increase in latency relative to sham blasted controls (SC) (51.3 s vs. 47.8 s; *p* = 0.0293). Whereas, blast exposed rats that received hyaluronidase infusion (HB) had a significantly decreased latency to platform when compared to saline treated blasted animals (SB) (44.0 s vs. 51.3 s.; *p* = 0.0326). Sham blasted control rats that received hyaluronidase infusion (HC) had similar MWM performance compared to their saline infused blast exposed counterparts (SB) (55.3 s vs. 51.3 s; *p* > 0.05). Furthermore, the HC treated group had significantly increased latency when compared to SC treated group (56.25 s vs. 46.69 s; *p* = 0.0259). Line graph depicts mean MWM trial distance over four consecutive blocks of trials **(B)**. Distance was significantly different between groups [One-way RM ANOVA: exposure *F*(3,45) = 6.331, *P* = 0.0011; time *F*(15,45) = 32.51, *P* = 0.0001]. HB rats took significantly shorter routes to the platform when compared to the SC group (890.9 cm vs. 1095 cm; *p* = 0.0247), the HC group (890.9 cm vs. 1183 cm, *p* = 0.0007), and the SB group (890.9 cm vs. 1083 cm, *p* = 0.0313). Line graph depicts mean MWM trial swim velocity over four consecutive blocks of trials **(C)** Swim velocity was significantly different between groups [One-way RM ANOVA: exposure *F*(2.355,35.32) = 7.291, *P* = 0.0014; time *F*(15,45) = 24.18, *P* < 0.0001]. HB rats swam significantly slower when compared to the SC group (20.53 cm/s vs. 22.81 cm/s; *p* = 0.0071), the HC group (20.53 vs. 23.15, *p* = 0.0006), and the SB group (20.53 vs. 22.52, *p* = 0.0389).

The observed changes in glycocalyx area were associated with significant changes in MWM performance (**Figure 6**). Latency was significantly different between groups [One-way RM ANOVA: exposure *F*(2.18,32.7) = 8.123, *P* = 0.001; time *F*(15,45) = 29.49, *P* = 0.0001] (**Figure 6A**). *Post hoc* Sidak testing demonstrated significant subgroup differences. Blast exposed rats that received hyaluronidase treatment (HB) had a significantly decreased latency to platform when compared to saline treated blasted animals (SB) (44.0 s vs. 51.3 s; *p* = 0.0326) such that they were not different from saline treated controls (SC). Control rats that received hyaluronidase (HC) had similarly impaired MWM performance compared to their saline blast exposed counterparts (SB) (55.3 s vs. 51.3 s; *p* > 0.05). Furthermore, the HC treated group had significantly increased latency when compared to SC treated group (56.25 s vs. 46.69 s; *p* = 0.0259). Distance was significantly different between groups [One-way RM ANOVA: exposure *F*(3,45) = 6.331, *P* = 0.0011; time *F*(15,45) = 32.51, *P* = 0.0001] (**Figure 6B**). *Post hoc* Sidak testing revealed significant subgroup differences. HB rats took significantly shorter routes to the platform when compared to the SC group (890.9 cm vs. 1095 cm; *p* = 0.0247), the HC group (890.9 cm vs. 1183 cm, *p* = 0.0007), and the SB group (890.9 cm vs. 1083 cm, *p* = 0.0313). There were no additional significant differences observed in the *post hoc* analysis.

Swim velocity was significantly different between groups [One-way RM ANOVA: exposure *F*(2.355,35.32) = 7.291, *P* = 0.0014; time *F*(15,45) = 24.18, *P* < 0.0001] (**Figure 6C**). *Post hoc* Sidak testing revealed significant subgroup differences. HB rats swam significantly slower when compared to the SC group (20.53 cm/s vs. 22.81 cm/s; *p* = 0.0071), the HC group (20.53 vs. 23.15, *p* = 0.0006), and the SB group (20.53 vs. 22.52, *p* = 0.0389). There were no additional significant differences observed in the *post hoc* analysis.

## Discussion

The brain vasculature is a known target for blast induced injury. Markers for this injury include complement activation (Dalle Lucca et al., 2012) and vascular permeability (Abdul-Muneer et al., 2013). Clinically, perivascular tau accumulation in the deep sulci of the brains of blast injured and concussed patients are consistent with damage to the brain vasculature (Goldstein et al., 2012). The salient finding from this study is that damage to the vascular endothelial glycocalyx is associated with cognitive impairment following exposure to repeated low intensity blast. These results show that blast exposure damages the vasculature and impairs cognition at lower intensities that those needed to damage the brain parenchyma (Ahlers et al., 2012). Additionally, this study is the first to demonstrate the novel finding that glycocalyx integrity is associated with downstream cognitive performance. Taken together these findings identify the endothelial glycocalyx as a discrete component of the brain vasculature damaged by low intensity blast exposures which is associated with downstream behavioral deficits.

To evaluate the effects of low-intensity blast on the endothelial glycocalyx, we used a previously characterized repeated whole body blast paradigm that produced a subtle learning decrement in the MWM without gross or histological signs of injury to the CNS or lung tissues (Ahlers et al., 2012). The blast exposures were generated using a pneumatic shock tube that induced a rapid increase in overpressure to 40 ± 5 kPa for 3 ms in the rats brain (Chavko et al., 2007). This blast exposure paradigm induced an increase in latency to platform in the MWM when compared to controls; these results were similar to previously reported decrements seen in this blast paradigm (Ahlers et al., 2012). It should be noted that the behavioral decrements in this study were subtle with all animals learning the task by the final trial. This is a challenge inherent to mTBI studies where more severe decrements in MWM are associated with injuries that are grossly observable. The behavioral findings in our study were also consistent with those of Saljo et al. (2011). That group used 30 kPa blast exposures and reported similar subtle deficits on the rate and retention of learning in the MWM task. Interestingly, that group also reported increased ICP following blast exposure, a finding which provides indirect evidence of the relationship between a vascular component and the observed behavioral decrement. This is in-line with the increased vascular injury markers (VEGF, vWF, AQP1, and AQP4) acutely seen in the peripheral blood of mice exposed to a single low-intensity blast (51.7 kPa) exposure (Ahmed et al., 2015). Additionally, electron microscopic studies conducted by our group demonstrated subclinical evidence of small vessel damage and dysfunction several months following repeated low-intensity blast exposure (Gama Sosa et al., 2014).

The current study used electron microscopy to directly observe a uniform partial degradation of the glycocalyx in all brain regions studied. This expands on indirect observations of glycocalyx shedding reported in animal models of severe TBI (Jepsen et al., 2014). It has been well established that glycocalyx damage can cause perivascular pathology to include edema (van den Berg et al., 2003) and persistent perturbations in cerebrovascular perfusion (Vogel et al., 2000). Furthermore, degraded low molecular weight hyaluronan (a primary structural component of the glycocalyx) can bind the CD44v10 receptor variant causing impaired vascular integrity via RhoA activation and the disruption of tight junction and adherens junction proteins (Lennon and Singleton, 2011). Reports of similar disruption of tight junction proteins (Shetty et al., 2014) and increased vascular permeability (Readnower et al., 2010) following low intensity blast exposure provide additional indirect supporting evidence of the glycocalyx damage described here. While this is a novel finding associated with low-intensity blast exposure, it does not demonstrate a causal link between glycocalyx damage and the observed MWM decrements in this model.

To establish a link between glycocalyx damage and MWM decrements, hyaluronidase was infused during the blast exposure paradigm to degrade damaged hyaluronan and stimulate regeneration of the glycocalyx. Hyaluronidase is used extensively in structural and functional studies and is known to reduce glycocalyx thickness by ∼40% while also simultaneously increasing vascular permeability (Zeng et al., 2012). Studies in mice showed that the glycocalyx regenerates with respect to thickness and permeability 5–7 days following hyaluronidase infusion in mice (Potter et al., 2009). In the present study, hyaluronidase induced a thickening of the glycocalyx irrespective of exposure compared to saline controls in all brain regions studied within 72 h of the final infusion. This represents a shortened recovery and a greater increase in thickness than previously reported in other tissue beds such as the cremaster muscle (Potter et al., 2009). Whether this is a unique property of the CNS or a rat specific finding warrants further investigation. The effect of the hyaluronidase infusion on MWM outcome, however, was striking with infusion reducing latency deficits in blasted animals and inducing latency deficits in sham exposed controls. Furthermore the hyaluronidase blast group (HB) showed significantly reduced distance traveled and swim velocity when compared to the other study groups. This supports the latency findings suggesting that the HB group had reduced latency due to taking a more efficient path to the platform. The reduced swim velocity is suggestive of a reduced anxiety phenotype during the MWM task; however, the MWM test is not designed to capture that endpoint. Further study with alternate behavioral tasks such as the elevated zero maze is warranted. Due to the fact that the tissue half-life of hyaluronidase in muscle is upward of 19 h it is possible that infusion of hyaluronidase altered the way the brain tissue interacted with the blast wave. This hypothesis was not tested in the current study, however, there was no direct evidence of gross pathological changes associated with hyaluronidase infusion. This is further supported by the observation of a lack of a swim velocity decrement in the hyaluronidase control group or the saline blast group when compared to saline controls. That observation provides evidence that neither the hyaluronidase infusion nor the blast exposure significantly impaired the ability of the rats to swim.

This finding is novel in two ways. First, the reduction of the MWM decrement in the hyaluronidase-treated blasted rats identifies a causal link between the blast-induced glycocalyx damage and the behavioral decrements. Second, the induction of a MWM decrement in the hyaluronidase-treated sham exposed rats demonstrates a definitive link between glycocalyx integrity and cognitive function in the absence of blast injury.

While the observations of both glycocalyx damage and hyaluronidase reversal of the blast-induced behavioral decrements suggest a glycocalyx role in the mechanism underlying low intensity blast-induced brain injury, these experiments also raise additional questions. In particular why does hyaluronidase induced glycocalyx thickening improves MWM performance in blasted animals and impair performance in sham blasted animals? Some insight may be gleaned from a study using hyaluronidase in a mouse model of subarachnoid hemorrhage (SAH) (McConnell et al., 2015). In this study hyaluronidase infusion 48 h after SAH reversed microcirculatory failure as defined as a decrease in red blood cell velocity. That study did not, however, observe decrements in microcirculatory function in control blood vessels treated with hyaluronidase. Glucose utilization in the microvasculature is known to be reduced following hyaluronidase infusion (Potter et al., 2009) which could explain the impaired performance associated with hyaluronidase infusion in sham controls. This does not explain then the improvement in MWM performance in the hyaluronidase infused blasted rats.

While mean decrements in glycocalyx thickness were associated with MWM performance, individual glycocalyx thickness did not significantly correlate with MWM performance (data not shown). These observations suggest there are additional factors linking glycocalyx integrity with MWM performance. Additional mechanistic studies targeting cerebrovascular function endpoints would likely clarify the link between glycocalyx damage and MWM performance decrements. Endpoints such as capillary transit time, glucose utilization, and functional capillary density are of particular interest, as they are directly tied to glycocalyx integrity, (Landsverk et al., 2012) as well as cerebral autoregulation and tissue perfusion (Jespersen and Ostergaard, 2012).

Repeated low intensity blast exposures at the intensities described here are frequently encountered during training or combat operations. Indeed, it is well documented that the military breacher population is at risk for neurocognitive impairment as a consequence of repeated blast exposures (Tate et al., 2013; Carr et al., 2015). This study introduces the glycocalyx as a potential novel target in the pathophysiology of neurocognitive impairment. Furthermore, glycocalyx injury is probable in a substantial proportion of blast associated mTBI patients. Further studies are warranted to determine whether the observed injuries to the glycocalyx have direct consequences on vascular function, and whether the damage is persistent.

## Author Contributions

AH was lead investigator for the study and participated in all aspects of the study including design, execution, data collection, analysis, manuscript preparation and editing. MM, HZ, and MS were involved with execution of the studies, data analysis, manuscript preparation, and editing. RM and SA were involved in all aspects of study design, planning, and administration. Additionally they were integral to data interpretation and manuscript preparation.

## Conflict of Interest Statement

The authors declare that the research was conducted in the absence of any commercial or financial relationships that could be construed as a potential conflict of interest.

## Footnotes

1 http://imagej.nih.gov/ij/
