# Corrigendum: Repeated Low Intensity Blast Exposure Is Associated With Damaged Endothelial Glycocalyx and Downstream Behavioral Deficits

**DOI:** 10.3389/fnbeh.2019.00251

**Published:** 2019-12-17

**Authors:** Aaron A. Hall, Mirian I. Mendoza, Hanbing Zhou, Michael Shaughness, Richard M. McCarron, Stephen T. Ahlers

**Affiliations:** Neurotrauma Department, Operational and Undersea Medicine, Naval Medical Research Center, Silver Spring, MD, United States

**Keywords:** blast, glycocalyx, behavioral deficits, traumatic brain injury, repeated low intensity

In the original article, there was an error. The membrane thickness for the mylar membranes used for the blast exposure was incorrectly reported. The corrected paragraph provides the proper units and reflects a blast overpressure range which takes into account a shift in membranes used during the course of the study. Experimentally determined pressures and ranges are added.

A correction has been made to the **Materials and Methods** section, subsection **Blast Overpressure Exposure**, paragraph one:

“Rats were exposed to blast overpressure using the Walter Reed Army Institute of Research (WRAIR) shock tube as described previously. The shock tube has a 305 mm circular diameter and is a steel tube comprised of a 0.76 m compression chamber that is separated from a 5.18 m expansion chamber. The compression and expansion chambers were separated by (0.05 or 0.075 mm) polyethylene Mylar^TM^ sheets (DuPontCo., Wilmington, DE, United States) that result in a peak pressure wave of (36.45 ± 2.32 kPa), with a mean duration of (3.78 ± 0.09 ms) and an impulse of (80.92 ± 4.41 kPa^*^ms); mean± standard error for *n* = 12 similar to previous reports (Ahlers et al., 2012).”

Eric M. Maudlin-Jeronimo was initially included as an author in the published article. Author Eric M. Maudlin-Jeronimo had corrections to the methods contained in this corrigendum identified after acceptance of the manuscript which did not carry forward into the final document. As a result, they wish to be removed from the publication. The author list has been updated accordingly.

The authors apologize for this error and state that this does not change the scientific conclusions of the article in any way. The original article has been updated.
